# Establishment and external validation of prognosis prediction nomogram for patients with distant metastatic intrahepatic cholangiocarcinoma: based on a large population

**DOI:** 10.1186/s12885-024-11976-6

**Published:** 2024-02-16

**Authors:** Chen Yuan, Shubing Zou, Kai Wang, Zhigang Hu

**Affiliations:** 1 Hepato-Biliary-Pancreatic Surgery Division, Department of General Surgery, The Second Affiliated Hospital of Nanchang University, 330006 Nanchang, China; 2 Jiangxi Provincial Clinical Research Center for General Surgery Disease, Nanchang, China; 3 Jiangxi Provincial Engineering Research Center for Hepatobiliary Disease, Nanchang, China; 4 East China Institute of Digital Medical Engineering, Shangrao, China

**Keywords:** Intrahepatic cholangiocarcinoma (ICC), Distant metastasis, Nomogram, Prognosis, Risk factors

## Abstract

**Background:**

Most patients with intrahepatic cholangiocarcinoma (ICC) have developed distant metastasis at the time of diagnosis, while there is rear related nomogram to predict the prognosis.

**Methods:**

Clinical data of patients pathologically diagnosed of ICC with distant metastasis were retrospectively collected from the Surveillance, Epidemiology, and End Results (SEER) database during 2005 to 2019. Finally, patients diagnosed as ICC in the Second Affiliated Hospital of Nanchang University from 2014 to 2019 were collected for external verification. All data were divided into training cohort and validation cohort in a ratio of 7:3. The nomogram was established based on independent prognostic factors using Cox univariate and multivariate analyses. The area under the receiver operating characteristic (ROC) curves (AUC), the calibration curve and the decision curve analysis (DCA) were used to determine the prediction accuracy of the nomogram.

**Results:**

This study finally included 572 ICC with distant metastasis patients, another 32 patients collected by the author’s hospital were used as external verification. Results showed that age, surgery, radiotherapy and chemotherapy were independent prognostic factors, and nomogram was established. The AUC of predicting 3, 6, 9-month overall survival were 0.866, 0.841 and 0.786. The ROC curves and calibration curves showed that the nomogram had good predictive accuracy, and DCA showed that the nomogram had good clinical applicability.

**Conclusions:**

The nomogram has good accuracy in predicting prognosis of DM-ICC patients, which would be of good significance to improve the prognosis of these patients.

## Introduction

Intrahepatic cholangiocarcinoma(ICC)is the second most common malignant tumor of liver. The overall prognosis of ICC is poor due to the malignant and metastatic potential [1–3]. ICC originates from the epithelial cells of the intrahepatic bile duct and the biological behavior is completely different from hepatocellular carcinoma [4, 5]. In recent years, the morbidity and mortality rates of ICC have increased worldwide [6–8]. The pathogenesis of ICC is complex, and intrahepatic cholangiolithiasis is considered as key factor [9, 10]. Due to the lack of typical clinical manifestations and early diagnostic methods, a majority of ICC patients are in the advanced stages when first diagnosed, such as distant metastases [11]. ICC is different from hepatocellular carcinoma and distant cholangiocarcinoma both in terms of biological behavior and treatment strategy [12]. As for ICC with DM (DM-ICC), comprehensive evaluation of the prognosis based on clinical features including treatment strategies might provide more evidence for clinicians to make the best treatment decisions. Therefore, a prognostic model is urgently needed, while there is currently rear related predictive model for the prognosis of ICC with distant metastasis patients.

The purpose of this study is to establish a prognostic nomogram for predicting the prognosis of ICC with distant metastasis patients. Clinical data from Surveillance, Epidemiology, and End Results (SEER) database were collected and were divided into training and internal validation cohort. Nomogram was constructed based on the training cohort, and were further evaluated based the internal and external validation cohorts. The established nomogram can provide truly individualized prognosis predictions and therefore guide the clinical decisions for ICC with distant metastasis patients.

## Patients and methods

### Data of patients

ICC with distant metastasis patients were enrolled from the Surveillance, Epidemiology, and End Results (SEER) database from the period of 2005 to 2019, by using the SEER∗Stat (National Cancer Institute, Bethesda, MD, USA) software version 8.4.0.1. The data were collected from the International Classification of Diseases for Oncology 3rd edition (ICD-O-3), primary site code C22.1 (intrahepatic bile duct), along with histological/behavior code 8160.3 (cholangiocarcinoma), and were randomly divided into training and validation cohort in a ratio of 7:3. The exclusion criteria were as follows: (1) Age younger than 18 years at diagnosis; (2) Combined with other primary tumors; (3) Incomplete clinical data or missing follow-up; (4) Postoperative survival less than 1 month. Institutional Review Board approval and informed consent are exempt because the SEER database is a public database with open access to anyone who has registered an account and signed a power of attorney.

### Statistical analysis

Continuous variables were expressed as mean ± standard deviation (SD) or median (range) and compared using the Mann-Whitney U test. Frequency variables were presented as numbers and percentages and were compared using the chi-square test or Fisher’s exact test when appropriate. Cox multivariate analysis was used to identify independent risk factors from which nomograms were constructed. The C-index and calibration curve were used to evaluate the accuracy of the nomogram [13]. In addition, we also plotted the receiver operating characteristic (ROC) curve and the area under the curve (AUC) to evaluate the accuracy of the nomogram. The decision curve analysis (DCA) identifies and compares clinical value between nomogram model and other clinical features by calculating the net benefit at each risk threshold probability [14–17]. All data were analyzed using R Studio 2022.07.0 + 548. Two-sided *P* < 0.05 was statistically significant.

## Result

### Patients characteristics

**Fig. 1:**
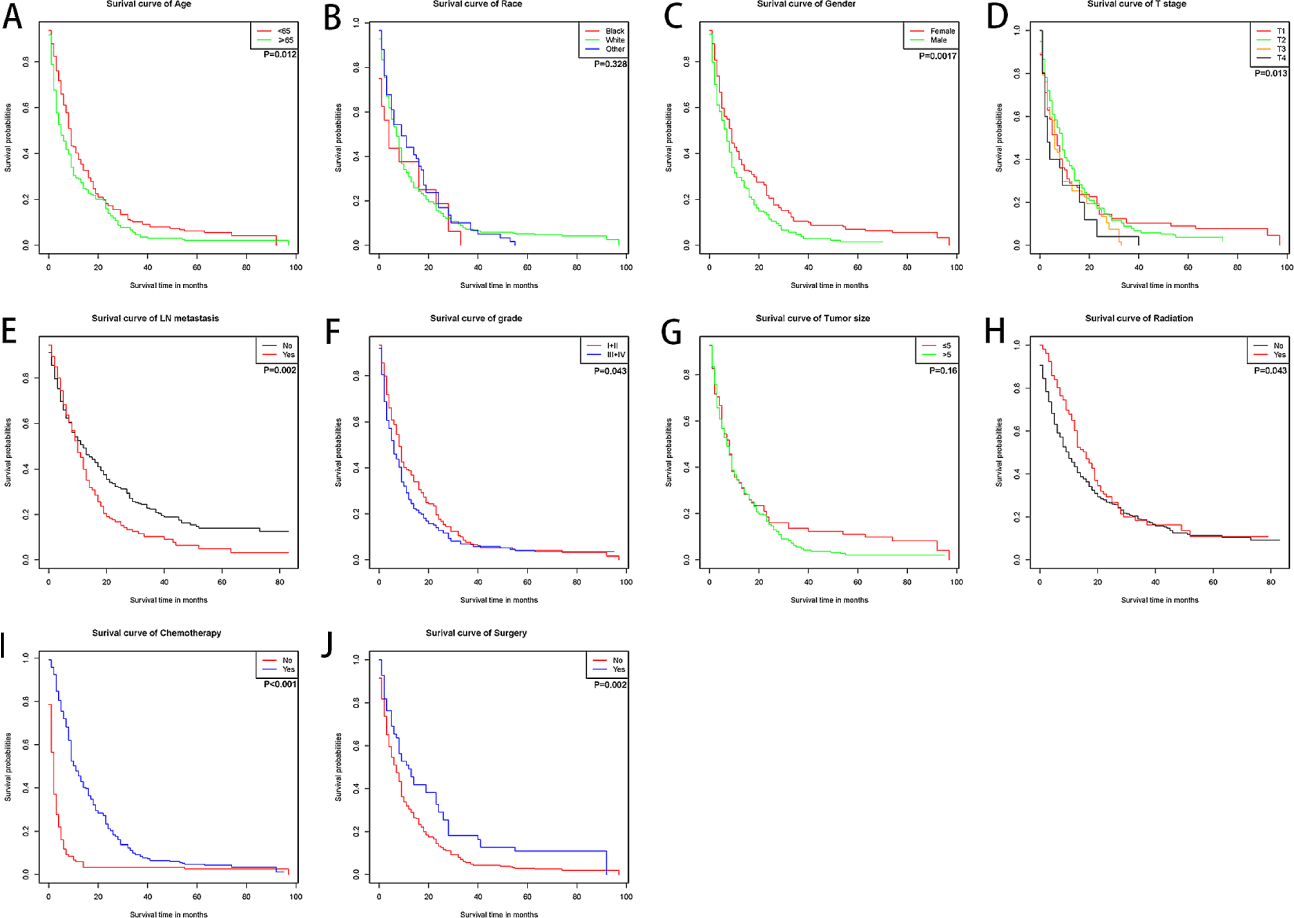
Overall survival rates according to patient characteristics: (A) Age; (B) Race; (C)Gender;(D) T stage;(E) LN metastasis; (F)Grade; (G)Tumor size; (H)Radiotherapy; (I)Chemotherapy;(J) Surgery. Abbreviation LN, lymph node

A total of 572 patients were included in this study, and were randomly divided into training and validation cohort by 7:3. There were 383 patients in the training cohort and 189 patients in the validation cohort, another 32 patients collected by the author’s hospital were used as external verification. The detail information about the training cohort and validation cohort were shown in Table 1a. The basic information of the training cohort and internal validation cohort divided by the SEER database is shown in Table 1b. The median survival for the overall data was 10 months. The Kaplan–Meier curves were drawn according to different clinical variables, and there were significant differences between the different variables, as shown in Fig. 1. Poorer prognosis is seen when patients are older than 65 years, have not undergone surgery, higher T stage, lymph node metastasis, higher grade, male gender and have not undergone chemoradiation therapy.

**Table 1a:** Clinical characteristics of patients in the SEER database and external validation cohort

**Characteristic**			**SEER cohort**		**Validation cohort**		
			**NO**	**%**	**NO**	**%**	
**Total**			572	100	32	100	
**Gender**		Female	246	43.0	16	50	
		Male	326	57.0	16	50	
**Race**		Black	28	4.9			
		White	466	81.5			
		Others	78	13.6			
**Age**		<65	288	50.3	19	59.4	
		≥ 65	284	49.7	13	40.6	
**Surgery**		No	498	87.1	8	25.0	
		Yes	74	12.9	24	75.0	
**Radiotherapy**		No	493	86.2	4	12.5	
		Yes	79	13.8	28	87.5	
**Chemotherapy**		No	178	31.1	5	15.6	
		Yes	394	68.9	27	84.4	
**T stage (8th)**		T1	146	25.5	4	12.5	
		T2	292	51.0	22	68.8	
		T3	98	17.1	2	6.2	
		T4	36	6.4	4	12.5	
**N stage**		N0	282	49.3	14	43.8	
		N1	290	50.7	18	56.2	
**Grade**		I + II	300	52.4	19	59.4	
		III + IV	274	47.6	13	40.6	
**Liver metastasis**		Absent	427	74.7	20	62.5	
		Present	145	25.3	12	37.5	
**Bone metastasis**		Absent	447	78.1	26	81.3	
		Present	125	21.9	6	28.7	
**Lung metastasis**		Absent	422	73.8	20	62.5	
		Present	150	26.2	8	37.5	
**Tumor size**		≤ 5 cm	129	22.6	9	28.1	
		>5 cm	443	77.4	23	71.9	

*LN* lymph node

**Table 1b:** Clinical characteristics of patients in the train cohort and internal validation cohort

Variable	Total (*n* = 572)	Train set (*n* = 400)	Valid set (*n* = 172)	Statistic	P
**Age**, n (%)				χ²=0.225	0.635
<65	288 (50.35)	204 (51.00)	84 (48.84)		
≥65	284 (49.65)	196 (49.00)	88 (51.16)		
**Gender**, n (%)				χ²=0.139	0.709
Female	326 (56.99)	230 (57.50)	96 (55.81)		
Male	246 (43.01)	170 (42.50)	76 (44.19)		
**Race**, n (%)				χ²=3.907	0.142
Black	28 (4.9)	23 (5.75)	5 (2.91)		
Others	78 (13.64)	49 (12.25)	29 (16.86)		
White	466 (81.47)	328 (82.00)	138 (80.23)		
**T stage**, n (%)				χ²=1.183	0.757
T1	146 (25.52)	101 (25.25)	45 (26.16)		
T2	292 (51.05)	202 (50.50)	90 (52.33)		
T3	98 (17.13)	69 (17.25)	29 (16.86)		
T4	36 (6.29)	28 (7.00)	8 (4.65)		
**N stage**, n (%)				χ²=1.537	0.215
N0	282 (49.3)	204 (51.00)	78 (45.35)		
N1	290 (50.7)	196 (49.00)	94 (54.65)		
**Surgery**, n (%)				χ²=1.664	0.197
No	498 (87.06)	353 (88.25)	145 (84.30)		
Yes	74 (12.94)	47 (11.75)	27 (15.70)		
**Radiotherapy**, n (%)				χ²=0.169	0.681
Yes	79 (13.81)	53 (13.25)	25 (14.53)		
No	493 (86.19)	347 (86.75)	147 (85.47)		
**Chemotherapy**, n (%)				χ²=0.777	0.378
No	178 (31.12)	120 (30.00)	58 (33.72)		
Yes	394 (68.88)	280 (70.00)	114 (66.28)		
**Tumor Size**, n (%)				χ²=0.012	0.911
>5 cm	443 (77.45)	311 (77.75)	133 (77.33)		
≤5 cm	129 (22.55)	89 (22.25)	39 (22.67)		
**Bone metastasis**, n (%)				χ²=0.265	0.606
No	447 (78.14)	317 (79.25)	133 (77.33)		
Yes	125 (21.86)	83 (20.75)	39 (22.67)		
**Liver metastasis**, n (%)				χ²=0.481	0.488
No	427 (74.65)	296 (74.00)	132 (76.74)		
Yes	145 (25.35)	104 (26.00)	40 (23.26)		
**Lung metastasis**, n (%)				χ²=0.154	0.694
No	422 (73.78)	297 (74.25)	125 (72.67)		
Yes	150 (26.22)	103 (25.75)	47 (27.33)		
**Grade**, n (%)				χ²=3.475	0.062
I + II	300 (52.45)	220 (55.00)	80 (46.51)		
III + IV	272 (47.55)	180 (45.00)	92 (53.49)		

### Independent risk factors of ICC with distant metastasis patients

The independent risk factors of patients with ICC with distant metastasis were determined by multivariate and univariate analysis in the training cohort, and the results are shown in Table 2. In multivariate analysis, age, surgery, chemotherapy, radiotherapy, T stage, lymph node metastasis, bone metastasis and grade were significantly correlated with the prognosis of patients with ICC with distant metastasis.

**Table 2:** Univariate and multivariate analyses for OS in patients of ICC with distant metastasis

Characteristic		Univariate analysis			Multivariate analysis		
		HR	95%CI	P*	HR	95%CI	P**
**Gender**	Female	Ref			Ref		
	Male	1.393	1.131–1.717	0.002	1.18	0.947–1.472	0.139
**Age**	<65	Ref			Ref		
	65	1.300	1.059–1.596	0.012	1.497	1.177–1.904	0.001
**Race**	Black	Ref			Ref		
	White	0.825	0.498–1.367	0.456	0.895	0.501–1.598	0.707
	Others	0.761	0.438–1.323	0.333	0.738	0.399–1.367	0.331
**Surgery**	No	Ref			Ref		
	Yes	0.629	0.466–0.847	0.002	0.544	0.391–0.758	< 0.001
**Radiotherapy**	No	Ref			Ref		
	Yes	1.289	1.018–1.539	0.04	0.489	0.342–0.713	< 0.001
**Chemotherapy**	No	Ref			Ref		
	Yes	0.284	0.225–0.359	< 0.001	0.191	0.146–0.250	< 0.001
**T stage**	T1	Ref			Ref		
	T2	1.021	0.767–1.274	0.418	1.130	0.861–1.480	0.373
	T3	1.272	1.109–1.653	0.046	1.558	1.112–2.185	0.009
	T4	1.486	1.192–1.814	0.012	2.119	1.255–3.577	0.004
**LN metastasis**	Absent	Ref			Ref		
	Present	1.318	1.010–1.519	0.028	1.253	0.798–0.964	0.047
**Bone Metastasis**	Absent	Ref			Ref		
	Present	1.287	1.479–1.841	0.041	1.440	1.016–2.041	0.040
**Liver Metastasis**	Absent	Ref			Ref		
	Present	1.254	0.993–1.583	0.058	1.255	0.899–1.752	0.183
**Lung Metastasis**	Absent	Ref			Ref		
	Present	1.533	1.210–1.943	< 0.001	1.247	0.905–1.718	0.177
**Grade**	I + II	Ref			Ref		
	III + IV	1.314	1.018–1.671	0.045	1.289	1.033–1.609	0.025
**Tumor size**	≤ 5 cm	Ref			Ref		
	>5 cm	1.198	0.927–1.548	0.168	1.138	0.839–1.545	0.406

* Cox regression analyses. OS, overall survival. ICC, intrahepatic cholangiocarcinoma. DM, distant metastasis. HR, hazard ratio. CI, confidence interval. LN, lymph node

### Development and validation of a nomogram for ICC with distant metastasis patients

**Fig. 2:**
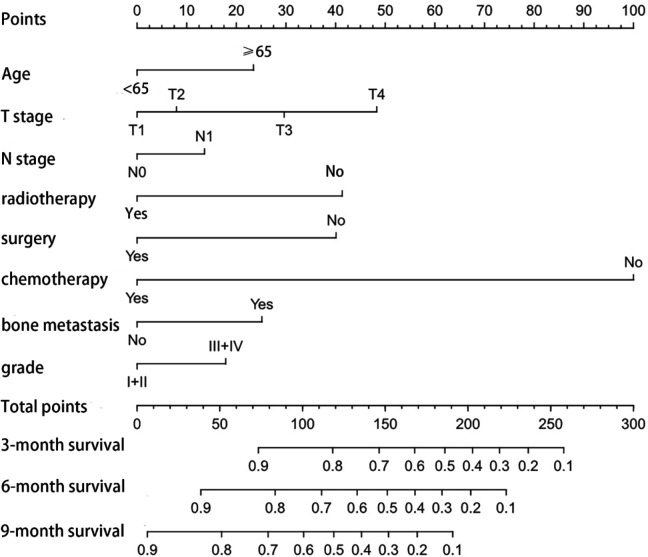
Nomogram predicting 3-,6-,9-month OS of ICC with distant metastasis patients. *Abbreviation* OS, overall survival

**Fig. 3:**
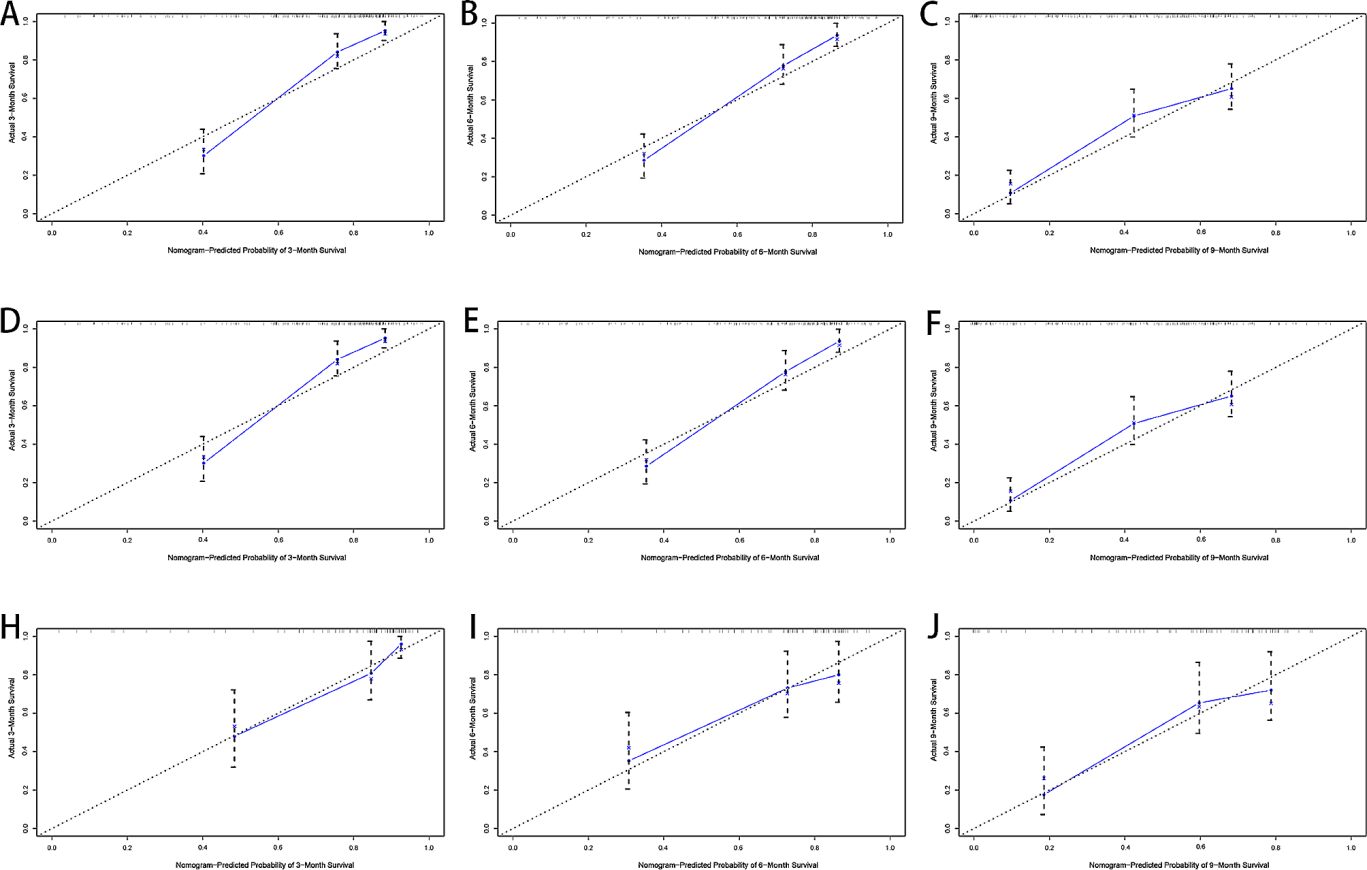
Calibration plots of the nomogram for 3-,6- and 9-month OS prediction of the training set (A, B, C), internal verification set (D, E, F) and external verification set (G, H, I). X-axis represents the nomogram-predicted probability of survival; Y-axis represents the actual OS probability. A perfectly accurate nomogram prediction model would result in a plot that the observed and predicted probabilities for given groups fall along the line. Dots with bars represent nomogram-predicted probabilities along with 95% confidence interval. Abbreviations OS, overall survival

In the training cohort, all factors that had a significant impact on the prognosis of patients with ICC with distant metastasis were included, and a nomogram was constructed, see Fig. 2. Through the superposition of the corresponding scores of different variables, the probability of different survival periods of the nomogram can be corresponded, which is very simple and easy to calculate. The C-index of the nomogram is 0.791, which can be concluded to have good prediction accuracy. In the training cohort as well as the validation cohort, the predicted survival rates for 3-, 6-, and 9-month prognosis were consistent with true survival (Fig. 3).

**Fig. 4:**
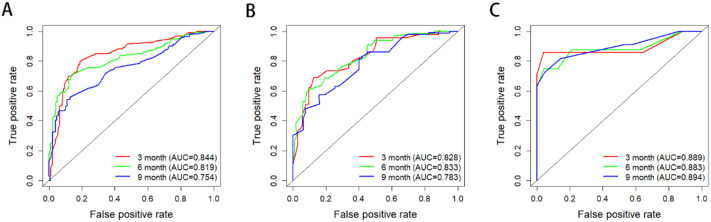
The ROC curves of the nomogram in the training set (**A**), the internal verification set (**B**) and external verification set (**C**) for 3-, 6- and 9-month OS prediction, *Abbreviations* OS, overall survival

**Fig. 5:**
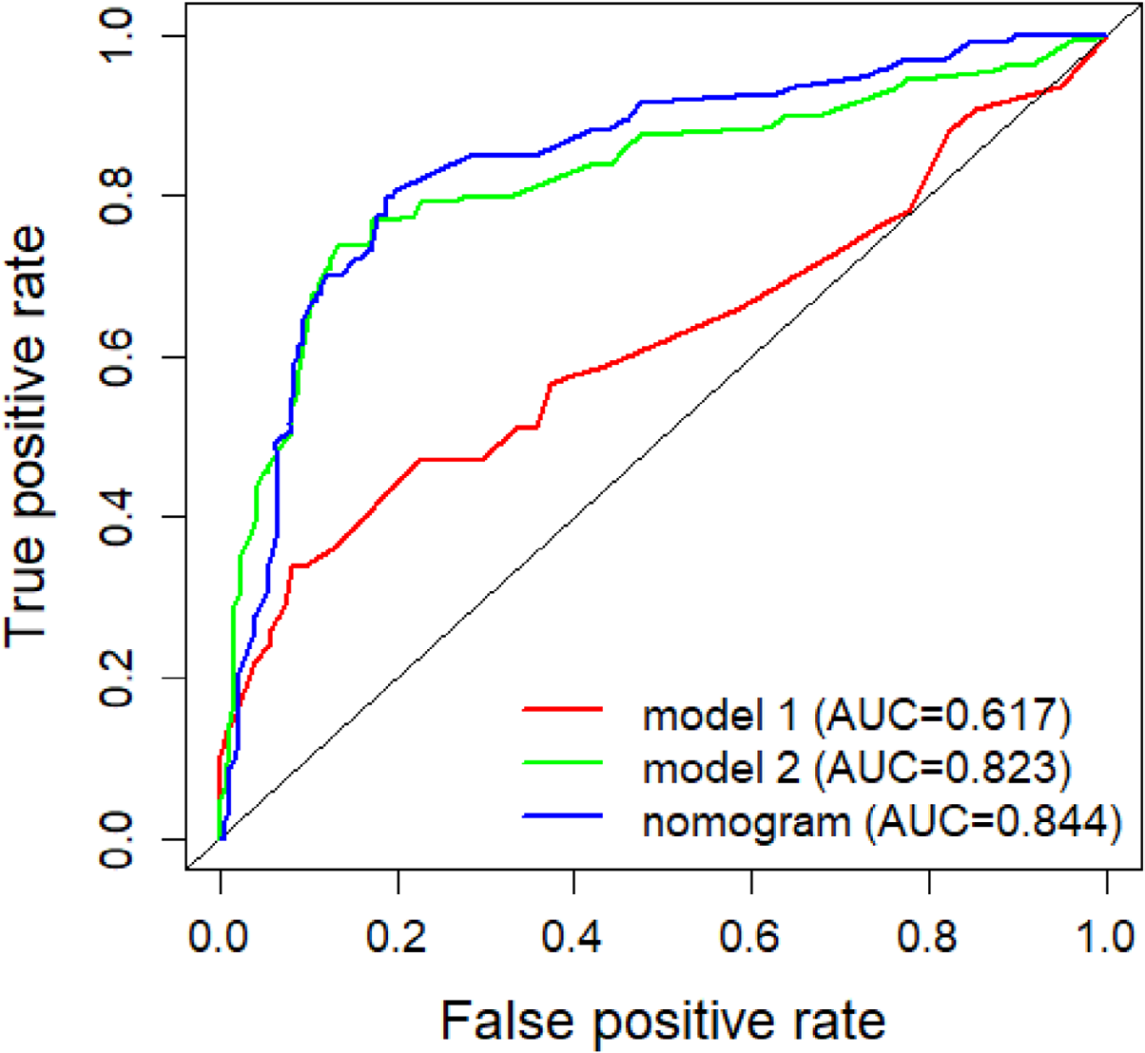
AUC comparison between different models. Model 1: All treatment options except for independent risk factors; Model 2: Excluding surgical treatment for independent risk factors

**Fig. 6:**
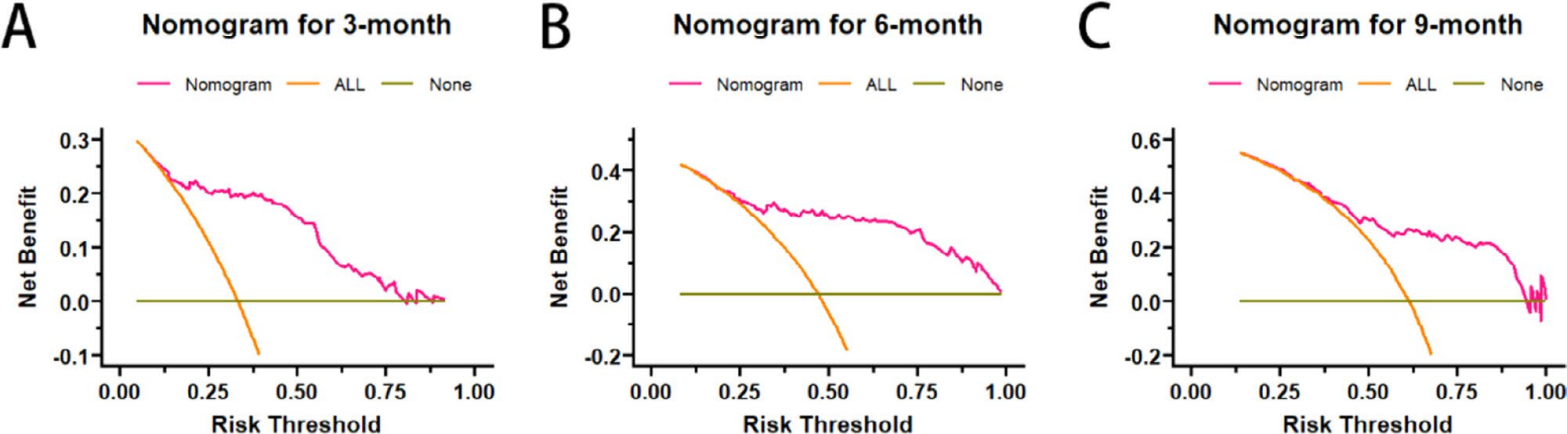
Decision curve analysis of nomograms a for predicting 3-month OS(**A**), 6-month OS(**B**), and 9-month OS(**C**)

Secondly, the accuracy of nomogram predictions was assessed by calculating the area under the ROC curve (AUC). In the nomogram, the AUC values for predicting prognosis in 3-, 6-, and 9-month were 0.844, 0.819, and 0.752, respectively (Fig. 4). The AUC of nomogram, internal validation and external validation are shown in Table 3. Then we compared the AUC of multiple factors. We found that different models have multicollinearity. We compared three models: Model 1 is an independent risk factor except for treatment, model 2 is an independent risk factor except for surgery, and model 3 is a nomogram (Fig. 5). Finally, the DCA curve was used to assess the clinical applicability of the nomogram, which were assessed by thresholds for each DCA curve (Fig. 6). From the graph, it can be seen that both the predicted DCA curves for 3-, 6-, and 9-month have a good threshold, so we can conclude that this nomogram has good clinical applicability. It can provide a better clinical decision for clinician.

**Table 3:** The AUC value of nomogram for predicting 3-month,6-month and 9-month OS

Patients	Overall survival		
	3-month	6-month	9-month
Training cohort	0.844	0.819	0.754
Internal validation cohort	0.828	0.833	0.783
External validation cohort	0.889	0.883	0.894

### Risk-stratified survival analysis based on nomogram

**Fig. 7:**
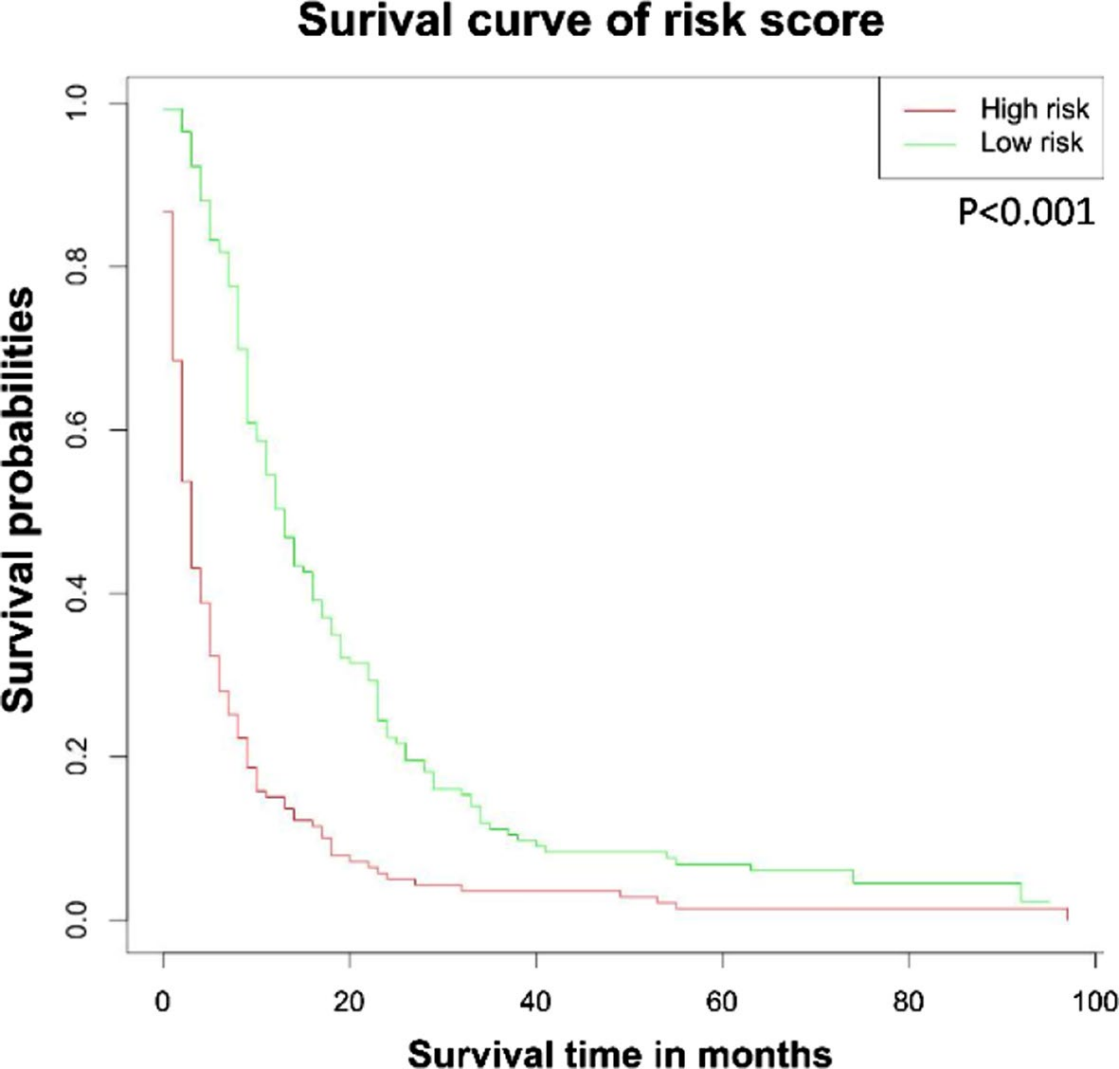
OS stratified by the risk levels of the nomogram-predicted survival probabilities. *Abbreviations* OS, overall survival

The probability scores for all patients were divided into two parts based on the mean. Patients with scores above the average were defined as high risk, and those with lower scores were defined as low risk. The survival rate of high-risk patients was significantly reduced as shown in Fig. 7 (*P* < 0.001).

## Discussion

It is necessary and attractive to develop a prognostic prediction model for patients with ICC with distant metastasis. Because it enables specific clinical decisions for different patients. In this study, for the first time, a prognostic prediction nomogram was constructed for patients with ICC with distant metastasis, and the internal validation was performed, and showed good prediction accuracy, which can provide different clinical decisions. Most previous studies [18–20] have constructed nomograms for predicting prognosis of postoperative ICC patients, but all of them included patients who were operable, and excluded patients who were inoperable. Therefore, most of the previous nomograms did not take into account advanced patients, especially those with distant metastasis. For clinicians, all patients cannot give up, and it is necessary and urgent to construct the required nomogram. Under this premise, we constructed a nomogram of patients with ICC of distant metastases with good predictive accuracy.

For most cancer patients, increasing age is an obvious factor affecting the prognosis of patients [21, 22]. As the patient’s age increases, the resistance to the tumor decreases and the sensitivity to the treatment drug decreases, the patient’s prognosis will show a significant decline, which can also be seen in our nomogram, age is an independent risk factors affecting patient prognosis. In our study of patients with ICC with distant metastasis, age, tumor differentiation, T stage, lymph node metastasis, surgical treatment, chemoradiotherapy, and bone metastasis had a significant impact on survival. As stated in the 8th TNM staging, higher TNM staging means worse prognosis, and in our study as well, higher T staging and patients with lymph node metastasis had worse prognosis. Higher T stage means deeper tumor infiltration, and lymph node metastasis usually affects the postoperative recovery of patients, increases the probability of tumor recurrence, and affects the prognosis of patients [23]. As in most previous studies [24, 25], the degree of tumor differentiation also affects patient outcomes. The higher the tumor differentiation, the stronger the malignant behavior of the tumor. Because the degree of differentiation reflects the malignant biological behavior of tumors, the lower the differentiation, the higher the degree of malignancy, and the higher the degree of differentiation, the lower the degree of malignancy. Because tumor cells arise from normal organs, the higher the differentiation, the closer the tumor is to normal cells, and the lower the nature and malignancy. Low or poorly differentiated tumors, especially undifferentiated tumors, indicate that the greater the difference from normal organs, the higher the degree of malignancy.

Until now, there are few studies on surgery for patients with ICC of distant metastases, and even fewer studies on surgery for liver resection and resection of metastases. Therefore, in this study, we included patients who underwent surgical treatment, and the operations performed were liver resection and metastases resection. Due to the large trauma of surgery and the advanced stage of the patient’s tumor, whether or not to perform surgery needs to be considered comprehensively, but surgery is still a means of treatment, which can prolong the survival of patients. Although the prognosis of ICC patients who undergo surgery is much better than that of patients who do not undergo surgery, most patients lose the opportunity for radical surgery at the time of diagnosis due to locally advanced or distant metastases [26–28]. It is more common in patients with ICC of distant metastases, usually accompanied by large blood vessel invasion or multiple metastases when accompanied by distant metastases, so there are fewer opportunities for surgical treatment. At present, radiotherapy, chemotherapy and targeted therapy have been accepted by clinicians as non-surgical treatment methods of tumors, so these treatment methods must be considered in the clinical treatment of tumors. In our study, radiotherapy and chemotherapy were considered as a treatment modality in the treatment of patients with ICC with distant metastasis, and the results showed that both radiotherapy and chemotherapy had the effect of prolonging the prognosis of patients, which was consistent with previous study [5]. Therefore, radiotherapy and chemotherapy can be used as a treatment for patients with advanced ICC, which can prolong the survival of patients.

At present, there is no prediction model for ICC patients with distant metastasis. However, in the nomogram we constructed, distant metastasis occurs. The prognosis of patients with bone metastases is significantly affected. A previous study [29] showed that the survival of patients with ICC of bone metastases was lower than with lung metastases and peritoneal metastases, but there was no statistical difference, and our results showed a difference, so far regardless of the proportion of metastases, the overall survival of patients^,^ period is still low. In conclusion, we constructed a prognostic prediction nomogram for patients with ICC of distant metastases, with good prediction accuracy, high C-index, and calibration defects not far from the actual values. The larger the C index, the more accurate the prognosis prediction to a certain extent [30]. However, high prognostic prediction accuracy does not necessarily imply good clinical applicability of nomogram [31]. The decision curve analysis uses an estimated threshold probability distribution and the weighted area under the net benefit curve as a summary metric to judge the clinical utility of the nomogram by the magnitude of the threshold [14, 32, 33].

Our study is the first to construct a nomogram in a patient with ICC of distant metastases, and external validation was performed. Of course, our study also has some limitations. First, the small number of external validation patients and the single external unit data may affect the validation accuracy of nomogram. Second, there is no relevant serological examination in the SEER database, and these variables will be included in our future studies. Additionally, similar to other retrospective studies, patient inclusion was subject to selection bias. Despite this limitation, we constructed a nomogram with good predictive accuracy and clinical applicability.

## Conclusion

Based on the SEER database, we constructed a nomogram for predicting ICC patients with distant metastasis in 3-, 6-, and 9 months. The nomogram has good prediction accuracy and clinical applicability, and can provide individual patients for different patients therapeutic strategies.

## Data Availability

The datasets generated or analyzed during this study are available from the corresponding author on reasonable request. SEER database data can be directly accessed and obtained from seer.cancer.gov.
